# Associations between gut microbiota and three prostate diseases: a bidirectional two-sample Mendelian randomization study

**DOI:** 10.1038/s41598-024-54293-5

**Published:** 2024-02-18

**Authors:** Xiaoyang Liu, Qiang Dong

**Affiliations:** Department of Urology, Institute of Urology, West China Hospital, Sichuan University, Chengdu, China

**Keywords:** Gut microbiota, Mendelian randomization, Prostatitis, Prostate cancer, Benign prostatic hyperplasia, Computational biology and bioinformatics, Microbiology, Urology

## Abstract

According to previous observational researches and clinical trials, the gut microbiota is related to prostate diseases. However, the potential association between gut microbiota and prostate disorders is still uncertain. We first identified groups of gut microbiota based on the phylum, class, order, family, and genus levels from consortium MiBioGen. And we acquired prostate diseases statistics from the FINNGEN study and PRACTICAL consortium. Next, two-sample Mendelian randomization was used to investigate the potential associations between three prevalent prostate disease and gut microbiota. In addition, we performed a reverse MR analysis and Benjamini-Hochberg (BH) test for further research. We investigated the connection between 196 gut microbiota and three prevalent prostate diseases. We identified 42 nominally significant associations and 2 robust causative links. Upon correction for multiple comparisons using the Benjamini–Hochberg procedure, our analysis revealed a positive correlation between the risk of prostatitis and the presence of the taxonomic order Gastranaerophilales. Conversely, the risk of prostate cancer exhibited an inverse correlation with the presence of the taxonomic class Alphaproteobacteria. Our study revealed the potential association between gut microbiota and prostate diseases. The results may be useful in providing new insights for further mechanistic and clinical studies of prostate diseases.

## Introduction

Any medical issue affecting the prostate gland, a tiny, walnut-shaped gland found in the male reproductive system, is referred to as prostate disease. Prevalent prostate illnesses include prostatitis, prostate cancer, and benign prostatic hyperplasia (BPH). Prostatitis is a frequent illness affecting the urinary tract and this inflammation can lead to various symptoms, including pain or discomfort in the pelvic region, difficulty urinating, and other urinary problems. Prostatitis can be classified into different types, with bacterial and non-bacterial forms being the main categories. Up to 16% of males in the US population are diagnosed to have symptomatic prostatitis in their lives^1,2^. Nevertheless, a considerable proportion of prostatitis cases present without overt symptoms, indicating a significant number of potential prostatitis patients may be going undetected. As the second greatest cause of cancer-related death globally, prostate cancer is the most frequent malignancy in males over the age of 60^3^. Despite the fact that the vast majority of patients are diagnosed with the inactive or very slow-progressing disease, it is predicted that around 20% of men with prostate cancer have high-risk cancer that will advance to a potentially deadly illness^3^. Benign prostatic hyperplasia, characterized by urinary retention and incontinence, is the most prevalent urologic condition in senior guys, affecting around 25% of men in their 50 s, 33% of men in their 60 s, and 50% of those in their 80 s^4,5^. This condition involves the proliferation of prostate cells, leading to an increase in gland size. BPH is a prevalent urological disorder, particularly affecting aging males, and it often gives rise to lower urinary tract symptoms (LUTS) due to the compression of the urethra by the enlarged prostate. In conclusion, prostate diseases are major health issues, especially for middle-aged and elderly men. Prostatitis, prostate cancer, and BPH can have serious health consequences, impacting urinary function and quality of life. The key to managing these conditions and minimizing their impact on health is early detection and treatment.

The gut microbiota, a vast and diverse community of microorganisms, performs a significant function in the host's physiology through the production of hormone-like substances, in addition to aiding in digestion and nutrient absorption^6^. Nowadays, the gut microbiota is now being considered a potential risk or protective factor for many different illnesses^7,8^. Microbiome dysbiosis is implicated in the development of prostate disorders in an increasing number of studies^9,10^. Shoskes found that patients with chronic prostatitis have substantially less diversity in their gastrointestinal microbiome, which aggregates differently compared to controls^11^. In Du's study, an experimental autoimmune prostatitis mouse model was developed to investigate whether gut microflora modulates Th17/Treg Cell differentiation via the short-chain fatty acid propionate, which may represent the pathogenic function of gut microbiota in prostatitis^12^. As for prostate cancer patients, more and more studies show that the gut microbiota of prostate cancers differs significantly from the normal group^13–16^. Pathways in folate and arginine metabolism were shown to be elevated in PCa patients using gene-based functional analysis of intestinal microbiota. Consequently, PCa cells became increasingly dependent on folate, and it has been demonstrated that blood folate levels are correlated with PCa risk and proliferation rate^17,18^. Matsushita also found that the short-chain fatty acids produced by gut microbes induce the release of IGF-1, which in turn affects prostate cancer development by stimulating local prostate MAPK and PI3K signalling, which also indicates the existence of a gut microbiota-IGF1-prostate axis^19^. The intestinal microbiota of men with enlarged prostates has been shown to have a larger proportion of Firmicutes to Bacteroidetes than that of individuals without^20^. Gu also discovered that BPH mice have the highest ratio of Firmicutes and Bacteroidetes induced by a high-fat diet, and the findings indicated that the gastrointestinal microbiota may be associated with Ghrelin, which is essential for the activation of Jak2/Stat3 in BPH progression^10^. However, the links between the intestinal microbiota and prostate diseases is still unclear and it is crucial to study the link between the gut's microbiome and prostate diseases.

Mendelian randomization (MR) analysis is a widely utilized technique for investigating possible links between environmental factors and illness because it exploits the intrinsic characteristics of common genetic variants for variable environmental exposures of interest^21–23^. Using two-sample MR analysis, researchers may unify disparate GWASs' SNP-exposure and SNP-outcome relationships into a single causal estimate. The availability of massive summaries of data on gut microbiota and illnesses has expanded with the proliferation of genome-wide association studies (GWASs), which have made two-sample statistical analysis employing MR far more reliable^24^.

In this study, Investigating the potential connections between prostate diseases and gut microbiota increases our knowledge of enteric bacterial pathogenesis and facilitates the development of dietary or microbiome-based personalized treatments for urinary system disorders. In order to determine the causal relationship between three prevalent prostate diseases and gut microbiota, two-sample MR analyses were performed in this study, which may contribute to providing new insights for further mechanistic and clinical studies of prostate diseases.

## Methods

### Data sources of gut microflora and prostate diseases

MiBioGen collaboration undertook the largest multi-ethnic study of host-genetics-microbiome relationships, from which the human gut microbiome genetic variations were produced^24,25^. This was a large-scale, multi-ethnic GWAS that integrated 16S ribosomal RNA gene sequencing profiles with genotyping data obtained from 18,340 participants across 24 cohorts in diverse geographical locations. The MiBioGen has standardized all procedures and protocols that participating cohorts must adhere to, encompassing microbiome data processing, genotype data processing, genome-wide association analyses, and meta-analyses. This standardization serves to uphold the reliability and authenticity of the dataset, allowing for a robust analysis of the potential associations and interactions between specific human genetic variations and the taxonomic diversity within the gut microbiota. The primary objective of this investigation was to elucidate the intricate interplay between autosomal human genetic variants and the composition of the gut microbiome. The dataset comprised 211 bacterial taxa and 15 microbial taxa without specific species names (unknown family or genus) were excluded^24^.

BPH (26,358 cases and 110,070 controls), and prostatitis (3299 cases and 110,070 controls) GWAS summary statistics were obtained from FinnGen consortium R8 release data^26^. PCa summary statistics (79,148 cases and 61,106 controls) conducted by PRACTICAL Consortium were derived from the UK biobank study^27,28^. Extensive information on the cohorts used, the genotypes, the outcome criteria, and the association test is available on the FinnGen webpage (https://www.finngen.fi/en) and PRACTICAL Consortium.

### Instrumental variables (IVs) selection

**Figure 1:**
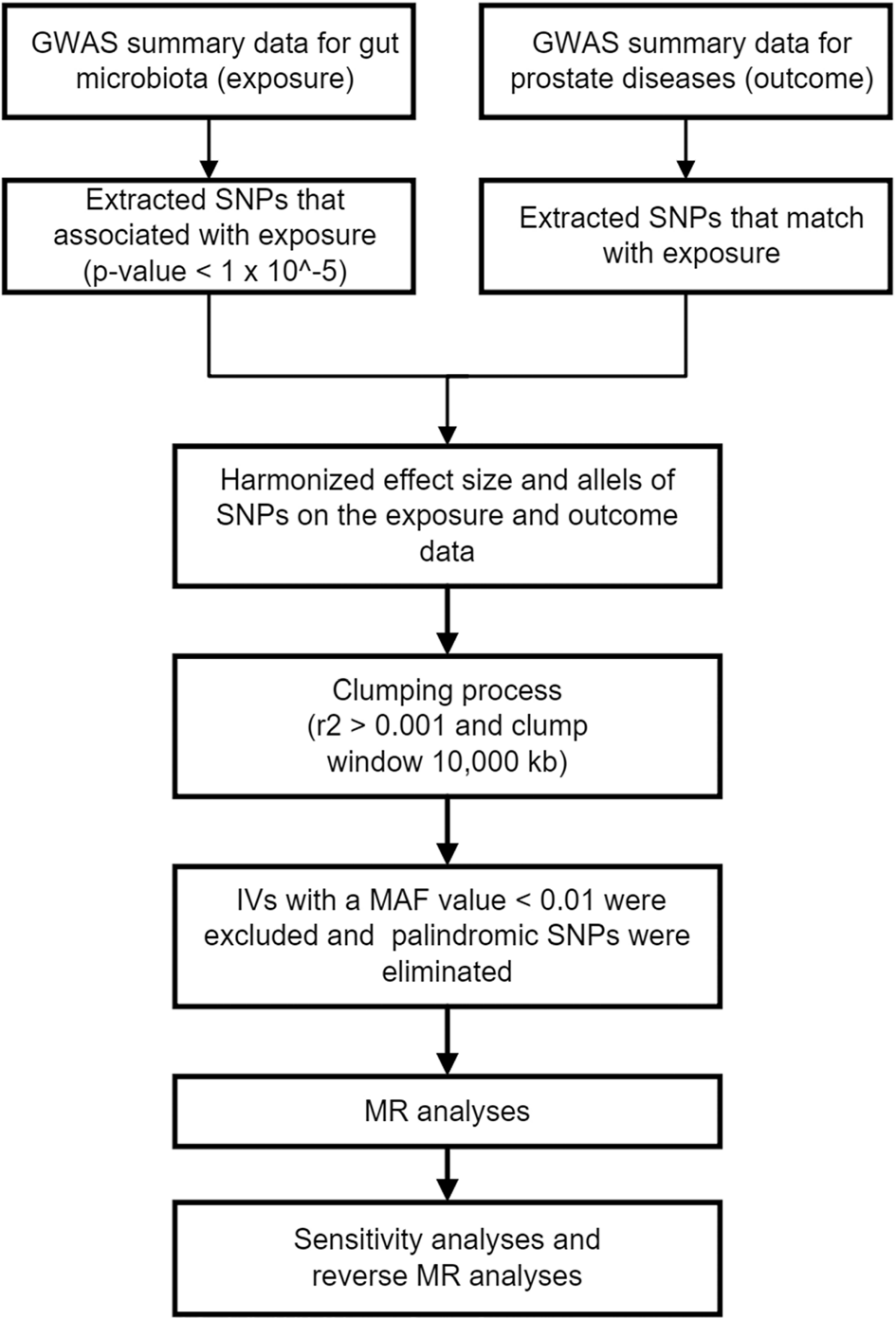
Flowchart of the study design and workflow.

The IVs were chosen based on the following criteria and the flowchart of our analysis is shown in Fig. 1. First, there must be a robust relationship between the exposure variables and the IVs selected for analysis. To attain a more comprehensive result, we chose IVs with a locus-wide significance level. Secondly, the IVs are required to pass the independence test. By excluding the SNPs using the PLINK clustering method (r2 > 0.001 and clump window 10,000 kb), the effect of linkage disequilibrium (LD) among the included genetic variants was avoided. Thirdly, any IVs with a MAF value < 0.01 are excluded. Lastly, during the harmonization procedure, we eliminated palindromic SNPs to guarantee that SNP effects on exposure are associated with the same allele as SNP effects on outcome. The selection criteria for IVs mentioned before ensures the validity of the outcomes of our study.

### Mendelian randomization

The association between exposure and outcome was studied using several different weighting schemes, including inverse variance weighted (IVW), MR-Egger, weighted median, simple mode, and weighted mode. Using the inverse-variance weighted (IVW) method, we estimated the Wald ratio for each genetic variant and then performed a summary. The IVW with multiplicative random effects method offers an accurate approximation and takes into consideration any possible variation between the Wald ratio estimations from SNPs^29^. The MR-Egger method is most useful for depicting the dose–response connection between IVs and outcomes while accounting for some pleiotropy^30^. The weighted median method takes into account the possibility of invalid genetic variations, which may reduce the occurrence of class 1 mistakes. Although some IVs do not meet the MR method's requirements for causal inference, the weighted mode approach is still credible when most IVs have similar causal estimations that are accurate. The robustness of the IVW technique results was further assessed using the simple and weighted modes^31^. If these methods produce different outcomes, we grant IVW priority as the primary outcome. To ensure that each IV was associated with the same effect allele, we harmonized the summary statistics and removed SNPs with ambiguous strands (A/T and C/G alleles). Eliminating palindromic SNPs prevented alleles from confounding the association between prostate diseases and gut microbiota taxa. Outliers and horizontal pleiotropy were examined using the MR-Egger and MR-PRESSO tests. In particular, MR-Egger was used to figure out whether horizontal pleiotropy existed with a p-value > 0.05 meaning no detectable horizontal pleiotropy.

Heterogeneity was evaluated by Conchrane's Q test^32^. Random-effects IVW models are used if there is heterogeneity, while the fixed-effect IVW model is used otherwise^33,34^. Using a leave-one-out sensitivity analysis, outliers and the validity of the findings were evaluated. The F-statistic, calculated by the formula F = R^2^ × (N-K-1)/(1–R^2^), was used to evaluate the efficacy of IVs^35^. Significant weak instrumental bias existed if the corresponding F-statistic was lower than 10. For a deeper comprehension of the link between cause and effect, Benjamini-Hochberg (BH) corrected p-values when they were deemed significant with a false discovery rate of less than 10%^36^. Nominal significance is defined as a p-value less than 0.05 or greater than the adjusted value. To further investigate this potential cause and effect, SNPs with a p-value < 1 × 10^–8^ were chosen for reverse causality analysis. All the statistical analyses were performed using R software version 4.2.2

## Results

### Instrumental variables (IVs) selection

Separately, we evaluated the IVs of 196 bacteria, comprising 119 genera, 32 families, 20 orders, 16 classes, and 9 phyl. After screening locus-wide significance level (p < 1 × 10^–5^) and the independence test (r2 > 0.001 and clump window 10,000 kb) according to the criteria mentioned above, a total of 2601 IVs were selected (Supplementary Material). We also excluded the IVs with F < 10 to guarantee that there is no significant weak instrumental bias.

### Two samples MR analysis of prostatitis

**Figure 2:**
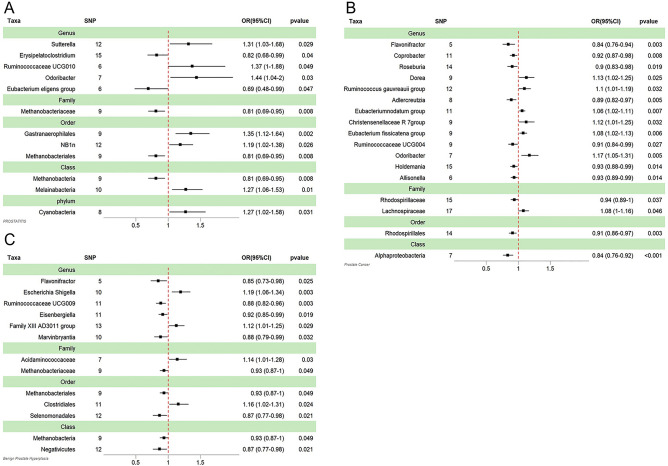
Mendelian randomisation results of causal effects between gut microbiome and cancer risk (p < 1 × 10^−5^). (**A**) Prostatis (**B**) Prostate cancer (**C**) Benign prostate hyperplasia.

Twelve associations between gut microbiota and prostatitis risk were discovered in our investigation after eliminating the Genus Ruminococcaceae UCG009 due to the existence of horizontal pleiotropy (MR-Egger intercept derived P-value 0.0198 < 0.05) (Fig. 2). In our analysis, Genus Odoribacter, Genus Ruminococcaceae UCG010, Genus Sutterella, Order Gastranaerophilales, Order NB1n, Class Melainabacteria, and Phylum Cyanobacteria were linked to an increased morbidity of prostatitis. Differently, Genus Eubacterium eligens group, Genus Erysipelatoclostridium, Family Methanobacteriaceae, Order Methanobacteriales, and Class Methanobacteria were linked to a decreased morbidity of prostatitis. For the remaining 12 exposures, neither the MR-Egger nor the MR-PRESSO analyses detected any horizontal pleiotropy or outliers (Table 1). In addition, according to the Cochrane Q test findings (Table 1), there is no significant heterogeneity among the chosen SNPs (p > 0.05). However, the leave-one-out analysis indicated that after removing the SNPs one by one, only the Class Methanobacteria, Order Gastranaerophilales, Order Methanobacteriales, and Family Methanobacteriaceae attained stable outcomes (Supplementary Fig. S4).

**Table 1:** Mendelian randomized outliers and level pleiotropy test of exposure and outcome.

Group	Exposure	Outcome	Cochran’s Q-derived P value	MR-PRESSO-global-test-derived P value	MR-Egger interceptderived P value
Genus	Sutterella	Prostatitis	0.25873355	0.3	0.295996303
	Erysipelatoclostridium	Prostatitis	0.84337329	0.856	0.287573032
	RuminococcaceaeUCG010	Prostatitis	0.13629342	0.214	0.87384877
	Odoribacter	Prostatitis	0.80425894	0.859	0.633613873
	Eubacteriumeligensgroup	Prostatitis	0.64698722	0.692	0.435812316
	Flavonifractor	Prostate cancer	0.09175144	0.157	0.661331618
	Coprobacter	Prostate cancer	0.63410591	0.685	0.305928271
	Roseburia	Prostate cancer	0.11739757	0.127	0.928997833
	Dorea	Prostate cancer	0.67488078	0.706	0.758672829
	Ruminococcusgauvreauiigroup	Prostate cancer	0.19925426	0.219	0.695495906
	Adlercreutzia	Prostate cancer	0.46751346	0.485	0.82581374
	Eubacteriumnodatumgroup	Prostate cancer	0.41688061	0.435	0.259850785
	Christensenellaceae R 7group	Prostate cancer	0.41663003	0.437	0.652945895
	Eubacteriumfissicatena group	Prostate cancer	0.06187809	0.075	0.503890653
	Ruminococcaceae UCG004	Prostate cancer	0.34953312	0.413	0.184899122
	Odoribacter	Prostate cancer	0.10355029	0.138	0.902354548
	Holdemania	Prostate cancer	0.20242788	0.229	0.409848885
	Allisonella	Prostate cancer	0.29559603	0.357	0.905234995
	Flavonifractor	BPH	0.3148249	0.365	0.731732647
	Escherichia Shigella	BPH	0.27472619	0.302	0.72509426
	Ruminococcaceae UCG009	BPH	0.06129275	0.058	0.746343621
	Eisenbergiella	BPH	0.39928593	0.415	0.305024255
	Family XIII AD3011 group	BPH	0.0544194	0.058	0.665181632
	Marvinbryantia	BPH	0.2263855	0.256	0.236552948
Family	Methanobacteriaceae	Prostatitis	0.89997696	0.911	0.497527832
	Rhodospirillaceae	Prostate cancer	0.07971029	0.097	0.271088881
	Lachnospiraceae	Prostate cancer	0.67299483	0.705	0.390626216
	Acidaminococcaceae	BPH	0.15931293	0.236	0.540354178
	Methanobacteriaceae	BPH	0.08607676	0.092	0.348266118
Order	Gastranaerophilales	Prostatitis	0.19563194	0.24	0.624139638
	NB1n	Prostatitis	0.7645008	0.79	0.468163293
	Methanobacteriales	Prostatitis	0.89997696	0.912	0.497527832
	Rhodospirillales	Prostate cancer	0.28928726	0.35	0.149372611
	Methanobacteriales	BPH	0.08607676	0.099	0.348266118
	Clostridiales	BPH	0.71227495	0.776	0.790653095
	Selenomonadales	BPH	0.67041302	0.72	0.707633178
Class	Methanobacteria	Prostatitis	0.89997696	0.915	0.497527832
	Melainabacteria	Prostatitis	0.10896208	0.148	0.364823074
	Alphaproteobacteria	Prostate cancer	0.22926616	0.289	0.780906172
	Methanobacteria	BPH	0.08607676	0.092	0.348266118
	Negativicutes	BPH	0.67041302	0.717	0.707633178
Phylum	Cyanobacteria	Prostatitis	0.10713617	0.155	0.470483497

### Two samples MR analysis of prostate cancer

Seventeen causal relationships between prostate cancer and gut microbiota have been demonstrated (Fig. 2). Genus Odoribacter, Genus Dorea, Genus Christensenellaceae R7 group, Genus Eubacterium fissicatena group, Genus Ruminococcus gauvreauii group, Genus Eubacterium nodatum group, and Family Lachnospiraceae are predicted to a higher risk of PCa whereas Genus Flavonifractor, Genus Adlercreutzia, Genus Roseburia, Genus Ruminococcaceae UCG004, Genus Coprobacter, Genus Allisonella, Genus Holdemania, Family Rhodospirillaceae, Order Rhodospirillales, and Class Alphaproteobacteria are predicted with a lower risk of PCa. There is no horizontal pleiotropy or outliers in terms of MR-Egger or MR-PRESSO results, and according to Cochrane’s Q test, there is also no obvious heterogeneity in the PCa analysis (Table S1). Furthermore, as shown in the leave-one-out analysis, the Genus Coprobacter, Class Alphaproteobacteria, Genus Adlercreutzia, and Order Rhodospirillales remain stable after excluding the SNPs one by one (Supplementary Fig. S5).

### Two samples MR analysis of benign prostate hyperplasia

This study uncovered thirteen underlying connections between BPH and gut microbiota (Fig. 2), including Genus Escherichia Shigella, Genus Family XIII AD3011 group, Family Acidaminococcaceae, and Order Clostridiales were predicted to a higher risk of getting BPH, while Genus Flavonifractor, Genus Marvinbryantia, Genus Ruminococcaceae UCG009, Genus Eisenbergiella, Family Methanobacteriaceae, Order Selenomonadales, Order Methanobacteriales, Class Negativicutes, and Class Methanobacteria were associated with a contrary effect. Also, neither the MR-Egger nor the MR-PRESSO tests detected any horizontal pleiotropy or outliers, and according to Cochrane’s Q test, there is no obvious heterogeneity in the BPH analysis (Table 1). However, after eliminating each SNP individually, the leave-one-out method revealed that only Genus Escherichia Shigella remained stable (Supplementary Fig. S6).

### Benjamini–Hochberg corrected test and reverse analysis

**Figure 3:**
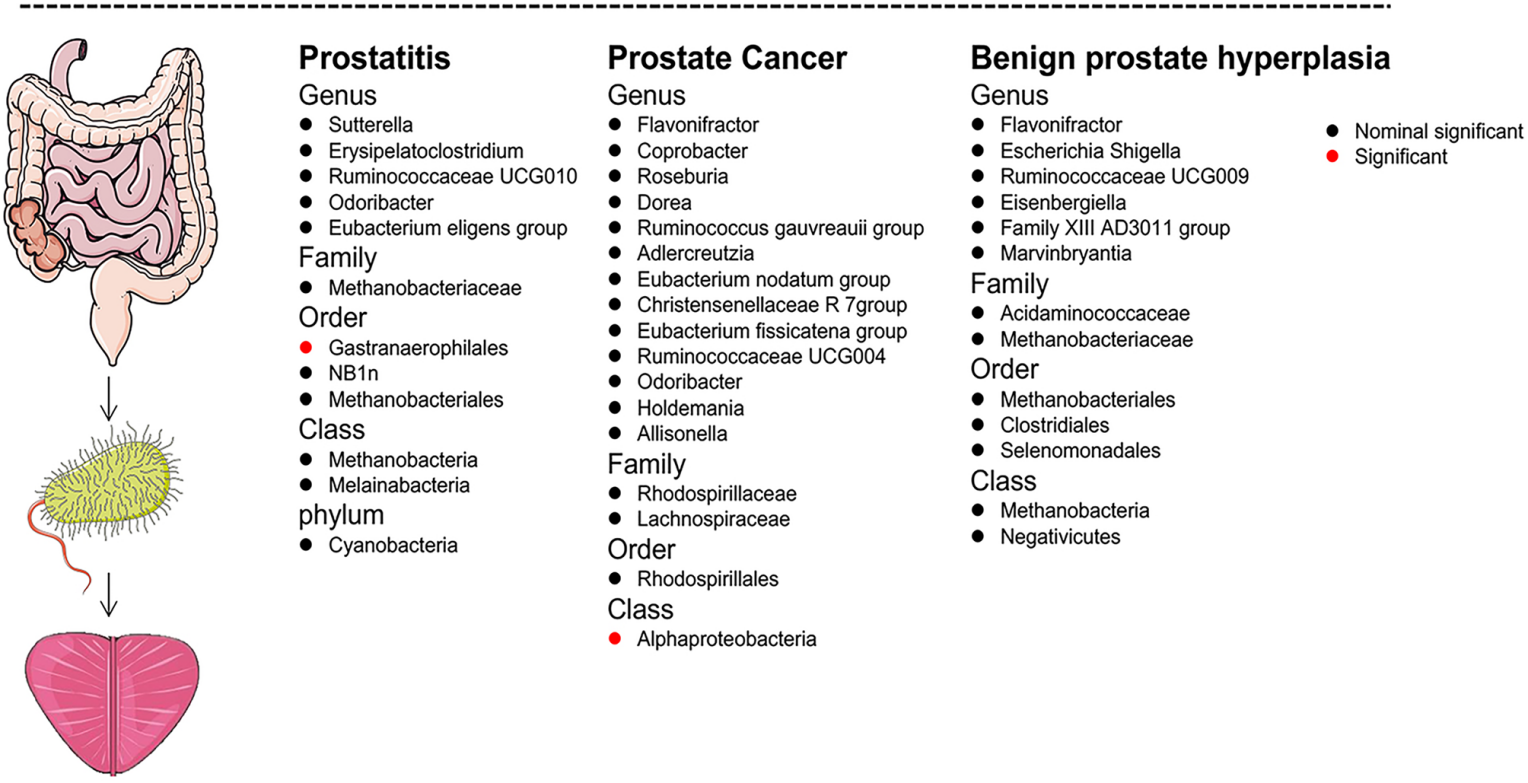
Nominal significant and significant associations between prostate disease and gut microflora.

There are 2 significant causal associations after BH corrected test (Fig. 3). Elevated Order Gastranaerophilales was found to be still linked with an increased risk of getting prostatitis (OR: 1.35, 95% CI 1.12–1.64, BH p-value: 0.0419) and elevated Class Alphaproteobacteria keeps a strong causal relationship with lower risk of PCa (OR: 0.84, 95% CI 0.76–0.92, BH p-value 0.002). Reversed analysis suggested that PCa was linked with an increased risk for Genus Flavonifractor (p = 0.049), but no such strong link was seen for other microbiota. (Supplement Table S3).

## Discussion

Our study represents the inaugural extensive and comprehensive Mendelian Randomization (MR) investigation within our current understanding, examining the potential association between gut microbiota and prostate diseases from a genetic vantage point. The biggest GWAS of the gut microbiome found robustly linked gene variations. We identified a genetic predisposition to gut bacteria that is causally linked to prostate illnesses such as prostatitis, prostate cancer, and benign prostatic hyperplasia. Overall, we were able to identify 42 distinct gut bacteria as potential risk factors for the aforementioned diseases. The results may be useful in providing new insights for further mechanistic and clinical studies of prostate diseases.

It was discovered that the gut microbiome's role in regulating inflammation, which is closely related to the development of prostatitis^11,37^. We successfully identified that Odoribacter, Ruminococcaceae UCG010, Sutterella, Gastranaerophilales, NB1n, Melainabacteria, and Cyanobacteria were linked with an elevated risk of getting prostatitis, while Eubacterium eligens group, Erysipelatoclostridium, and Methanobacteriaceae were linked with a lower risk of getting prostatitis. Previous studies have reported that in rats with chronic prostate inflammation there was a significant increase of Odoribacter, which confirms our study's results^38^. It has also been explored that Ruminococcaceae UCG010 and Sutterella might serve as driving factors in the progression of the diabetes, which may later lead to prostatitis due to a decrease in the function of autoimmune defense^39–41^. Previous research has also shown a link between cyanobacteria and gastrointestinal diseases such as adenomas and acute gastroenteritis^42^. Eubacterium eligens group, a beneficial bacteria, significantly correlated positively with CD3 + T cells and negatively with NK cells, which may take a toll in the prevention of prostatitis^43^. Also, there is growing evidence showing the Erysipelatoclostridium plays in immune response, inflammation, and response to cancer immune therapy^44^. Methanobacteriaceae is the predominant methanogen in the human gut, and its presence or absence in the gastrointestinal tract has been linked to a variety of health characteristics, including changes in body weight and cardiovascular disease^45^.

Researchers have looked into the link between the gut microbiota and cancer, and their results suggest that the microbiota may play an important role in the development and progression of malignant prostate cancer^46,47^. Using the mendelian randomization, our study demonstrated that Odoribacter, Dorea, Christensenellaceae R7 group, Ruminococcus gauvreauii group, Eubacterium fissicatena group, Eubacterium nodatum group, and Lachnospiraceae are associated with an increased risk of prostate cancer while Flavonifractor, Adlercreutzia, Roseburia, Ruminococcaceae UCG004, Coprobacter, Allisonella, Rhodospirillaceae, Holdemania, and Alphaproteobacteria are linked with a lower risk of prostate cancer. In the study of Xu^48^, Odoribacter was suggested to have significant heritability estimates (0.476), which may serve as a genetic carcinogenic factor for prostate cancer. In the research on the role of gut microbiome in predicting response to neoadjuvant chemoradiotherapy, Dorea, as a microbe related to butyrate production, was overrepresented in responders noticed in the baseline samples, which may be correlated with butyrate function in regulation of oxidative stress, maintenance of mucosal integrity, and reduction of inflammation via macrophage function^6,49^. Emerging studies have indicated that Christensenellaceae R7 group, Ruminococcus gauvreauii group, Eubacterium fissicatena group, and Eubacterium nodatum group are all associated with metabolism. In the study of Jian^50^, after low-energy diets, Christensenellaceae R-7 group was significantly increased and Verheggen^51^ found the abundance of Ruminococcus gauvreauii group significantly increased after an 8‐week aerobic exercise intervention. Enhancing the abundance of intestine anxiolytic and antidepressant short-chain fatty acids, Lachnospiraceae was found to be significantly rich in the HA (astaxanthin) treated prostate tumor, indicating a correlation between prostate cancer and microbiota^52^. An^53^ found BPH induction altered the abundance of Flavonifractor, but the relationship with prostate cancer requires further study. Consistent with our study, Adlercreutzia was found to have a decreased abundance in the high fat diet mice group, which has been shown to promote prostate carcinogenesis by influencing the gastrointestinal microbiota and microbiota-mediated equol metabolism adversely^54^. According to a cross-sectional research, patients with prostate cancer who had prostatectomy or androgen deprivation treatment had considerably reduced levels of Roseburia and alpha-diversity in their gut microbiota, which also confirmed our results. Although there is limited research in investigating the role of Ruminococcaceae UCG004, Coprobacter, Allisonella, Rhodospirillaceae, Holdemania in prostate cancer, these bacteria were verified in the correlation with colorectal cancer and metabolism, and indicated that their roles in immune function, inflammation, and hormone levels, all investigated to be linked with PCa development and progression^55–58^.

In this study, thirteen gut microbiota were found to be associated with benign prostate hyperplasia. Escherichia Shigella, XIII AD3011 group, and Acidaminococcaceae, Clostridiales were predicted to an increased risk of getting BPH, while Flavonifractor, Marvinbryantia, Ruminococcaceae UCG009, Eisenbergiella, Methanobacteriaceae, Selenomonadales, Methanobacteriales, Negativicutes, and Methanobacteria were on the contrary. Both the direct and indirect impacts of the gut microbiome on the androgens have been demonstrated in animal studies, which contribute to benign prostatic hyperplasia and Escherichia Shigella was found to be correlated with raised testosterone^59,60^. Consistent with our study, Clostridiales can convert primary bile acids into toxic secondary bile acids and convert glucocorticoids into androgens by side-chain cleavage, which is likely to contribute to progression of benign prostate hyperplasia^61^. In the study of An^53^, BPH induction altered the abundance of Flavonifractor, and Flavonifractor coule be a potential indicator for the diagnosis of BPH. Although there are limited studies investigating other gut microbiota and prostate or androgen levels, these gut microbiota are mostly found in the correlation of colorectal cancer metabolism, immune function and anti- inflammation^14^. Further studies on these gut microbiota are highly needed.

Notably, the BH test indicated that elevated Order Gastranaerophilales keeps a stable causal association with increased risk of prostatitis (OR: 1.35, 95% CI 1.12–1.64, BH p-value: 0.0419) and elevated Class Alphaproteobacteria keeps a stable causal association with decreased risk of prostate cancer (OR: 0.84, 95% CI 0.76–0.92, BH p-value 0.002). It has been shown that the bacteria Gastranaerophilales are crucial for Indole production, which in turn increases the synthesis of an anti-inflammatory and cancer-inhibiting actions substance-indolepropionic acid^62^. Consistent with our study, the study of Wei^63^ found that PCa risk decreased in correlation with increased levels of Alphaproteobacteria. And it's possible that lifestyle factors like overeating, drinking, and smoking are what really drive the protective effect of a high abundance of Class Alphaproteobacteria on PCa progress. Sookoian found that non-morbidly obese controls had a greater abundance of Alphaproteobacteria than morbidly obese patients, indicating that these bacteria might contribute to the development of PCa^64^. However, further study is required to completely comprehend the relationships between Alphaproteobacteria and PCa.

We also admit that there are certain limitations in our research. First, we selected all the SNPs that met the locus-wide significance level (1 × 10^−5^) because the SNPs obtained (5 × 10^−8^) were insufficient for further study. Second, the gut microbiome GWAS meta-analysis did not just include male participants. Although chromosomal regions associated with male and female characteristics were disregarded, potential sex-based bias remained. Finally, since the MR analysis is based on an untestable assumption, additional experimental and clinical validation is necessary to determine whether or not particular microbial species have any discernible effect on humans.

## Conclusions

Using the Mendelian randomization technique, we investigated the connection between 196 gut microbes and three prevalent prostate diseases and identified 42 nominally significant associations and 2 robust causative links. Upon correction for multiple comparisons using the Benjamini–Hochberg procedure, our analysis revealed a positive correlation between the risk of prostatitis and the presence of the taxonomic order Gastranaerophilales. Conversely, the risk of prostate cancer exhibited an inverse correlation with the presence of the taxonomic class Alphaproteobacteria. The study may be useful in providing new insights for further mechanistic and clinical studies of prostate diseases.

### Supplementary Information

Supplementary Information.

## Data Availability

All the statistics can be found in FINNGEN (https://www.finngen.fi/en) and PRACTICAL Consortium (http://practical.icr.ac.uk/blog/).
