# Supplementary material for: Associations between gut microbiota and three prostate diseases: a bidirectional two-sample Mendelian randomization study
Source: Sci Rep. 2024 Feb 18;14:4019. doi: 10.1038/s41598-024-54293-5 (PMC10874943; doi:10.1038/s41598-024-54293-5)
Supplement: Supplementary file 1 — Supplementary Information. [file 41598_2024_54293_MOESM1_ESM.docx]

***Supplementary Material***

**Associations between gut microbiota and three prostate diseases: A bidirectional two-sample Mendelian randomization study**

**Xiaoyang Liu^1^ Dong Qiang***

1Department of Urology, Institute of Urology, West China Hospital, Sichuan University, Chengdu, China

* Correspondence: Department of Urology, Institute of Urology, West China Hospital, Sichuan University, Chengdu, China dongqiang@scu.edu.cn

| **exposure** | **nsnp** | **or** | **or_lci95** | **or_uci95** | **pval** | **p_BH** |
| --- | --- | --- | --- | --- | --- | --- |
| **genus** |  |  |  |  |  |  |
| Sutterella | 12 | 1.31 | 1.03 | 1.68 | 0.03 | 0.76 |
| Erysipelatoclostridium | 15 | 0.82 | 0.68 | 0.99 | 0.04 | 0.76 |
| RuminococcaceaeUCG010 | 6 | 1.37 | 1.00 | 1.88 | 0.05 | 0.76 |
| Odoribacter | 7 | 1.44 | 1.04 | 2.00 | 0.03 | 0.76 |
| Eubacteriumeligensgroup | 6 | 0.69 | 0.48 | 0.99 | 0.05 | 0.76 |
| **family** |  |  |  |  |  |  |
| Methanobacteriaceae | 9 | 0.81 | 0.69 | 0.95 | 0.01 | 0.26 |
| **order** |  |  |  |  |  |  |
| Gastranaerophilales | 9 | 1.35 | 1.12 | 1.64 | <0.01 | 0.04 |
| NB1n | 12 | 1.19 | 1.02 | 1.38 | 0.03 | 0.18 |
| Methanobacteriales | 9 | 0.81 | 0.69 | 0.95 | 0.01 | 0.08 |
| **class** |  |  |  |  |  |  |
| Methanobacteria | 9 | 0.81 | 0.69 | 0.95 | 0.01 | 0.08 |
| Melainabacteria | 10 | 1.27 | 1.06 | 1.53 | 0.01 | 0.08 |
| **phylum** |  |  |  |  |  |  |
| Cyanobacteria | 8 | 1.27 | 1.02 | 1.58 | 0.03 | 0.28 |

**Supplement Table S1. Sensitivity analysis and BH test for prostatitis (fix effect model)**

| **exposure** | **nsnp** | **or** | **or_lci95** | **or_uci95** | **pval** | **p_BH** |
| --- | --- | --- | --- | --- | --- | --- |
| **genus** |  |  |  |  |  |  |
| Flavonifractor | 5 | 0.84 | 0.76 | 0.94 | <0.01 | 0.16 |
| Coprobacter | 11 | 0.92 | 0.87 | 0.98 | 0.01 | 0.16 |
| Roseburia | 14 | 0.90 | 0.83 | 0.98 | 0.02 | 0.26 |
| Dorea | 9 | 1.13 | 1.02 | 1.25 | 0.02 | 0.29 |
| Ruminococcusgauvreauiigroup | 12 | 1.10 | 1.01 | 1.19 | 0.03 | 0.29 |
| Adlercreutzia | 8 | 0.89 | 0.82 | 0.97 | 0.01 | 0.16 |
| Eubacteriumnodatumgroup | 11 | 1.06 | 1.02 | 1.11 | 0.01 | 0.16 |
| ChristensenellaceaeR.7group | 9 | 1.12 | 1.01 | 1.25 | 0.03 | 0.29 |
| Eubacteriumfissicatenagroup | 9 | 1.08 | 1.02 | 1.13 | 0.01 | 0.16 |
| RuminococcaceaeUCG004 | 9 | 0.91 | 0.84 | 0.99 | 0.03 | 0.29 |
| Odoribacter | 7 | 1.17 | 1.05 | 1.31 | <0.01 | 0.16 |
| Holdemania | 15 | 0.93 | 0.88 | 0.99 | 0.01 | 0.21 |
| Allisonella | 6 | 0.93 | 0.89 | 0.99 | 0.01 | 0.21 |
| **family** |  |  |  |  |  |  |
| Rhodospirillaceae | 15 | 0.94 | 0.89 | 1.00 | 0.04 | 0.58 |
| Lachnospiraceae | 17 | 1.08 | 1.00 | 1.16 | 0.05 | 0.58 |
| **order** |  |  |  |  |  |  |
| Rhodospirillales | 14 | 0.91 | 0.86 | 0.97 | <0.01 | 0.06 |
| **class** |  |  |  |  |  |  |
| Alphaproteobacteria | 7 | 0.84 | 0.76 | 0.92 | <0.01 | <0.01 |

**Supplement Table S2. Sensitivity analysis and BH test for prostate cancer (fix effect model)**

| **exposure** | **nsnp** | **or** | **or_lci95** | **or_uci95** | **pval** | **p_BH** |
| --- | --- | --- | --- | --- | --- | --- |
| **genus** |  |  |  |  |  |  |
| Flavonifractor | 5 | 0.85 | 0.73 | 0.98 | 0.02 | 0.64 |
| Escherichia.Shigella | 10 | 1.19 | 1.06 | 1.34 | <0.01 | 0.21 |
| RuminococcaceaeUCG009 | 11 | 0.88 | 0.82 | 0.96 | <0.01 | 0.21 |
| Eisenbergiella | 11 | 0.92 | 0.85 | 0.99 | 0.02 | 0.64 |
| FamilyXIIIAD3011group | 13 | 1.12 | 1.01 | 1.25 | 0.03 | 0.64 |
| Marvinbryantia | 10 | 0.88 | 0.79 | 0.99 | 0.03 | 0.64 |
| **family** |  |  |  |  |  |  |
| Acidaminococcaceae | 7 | 1.14 | 1.01 | 1.28 | 0.03 | 0.79 |
| Methanobacteriaceae | 9 | 0.93 | 0.87 | 1.00 | 0.05 | 0.79 |
| **order** |  |  |  |  |  |  |
| Methanobacteriales | 9 | 0.93 | 0.87 | 1.00 | 0.05 | 0.31 |
| Clostridiales | 11 | 1.16 | 1.02 | 1.31 | 0.02 | 0.24 |
| Selenomonadales | 12 | 0.87 | 0.77 | 0.98 | 0.02 | 0.24 |
| **class** |  |  |  |  |  |  |
| Methanobacteria | 9 | 0.93 | 0.87 | 1.00 | 0.05 | 0.39 |
| Negativicutes | 12 | 0.87 | 0.77 | 0.98 | 0.02 | 0.33 |

**Supplement Table S3. Sensitivity analysis and BH test for benign prostatic hyperplasia (fix effect model)**


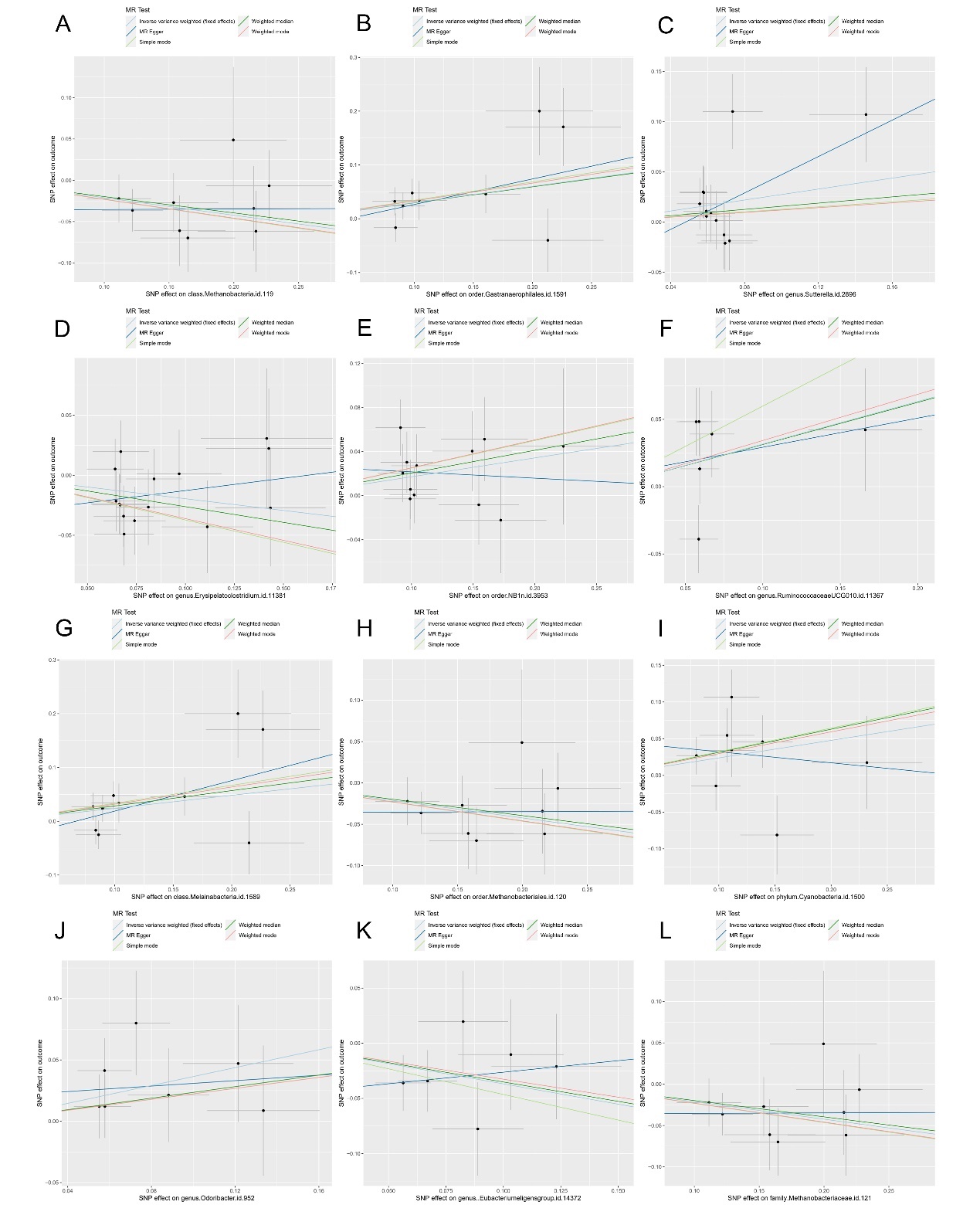


**Supplement Figure S1**

Scatter plots of significant and nominal significant estimates from genetically predicted

microbiotas(A) Family Methanobacteriaceae; (B) Order Gastranaerophilales; (C) Genus Sutterella; (D) Genus Erysipelatoclostridium; (E) Order NB1n ; (F) Genus RuminococcaceaeUCG010; (G) Class Melainabacteria; (H) Order Methanobacteriales; (I) Phylum Cyanobacteria;(J) Genus Odoribacter;(K) GenusEubacterium eligens group;(L) Family Methanobacteriaceae on prostatitis


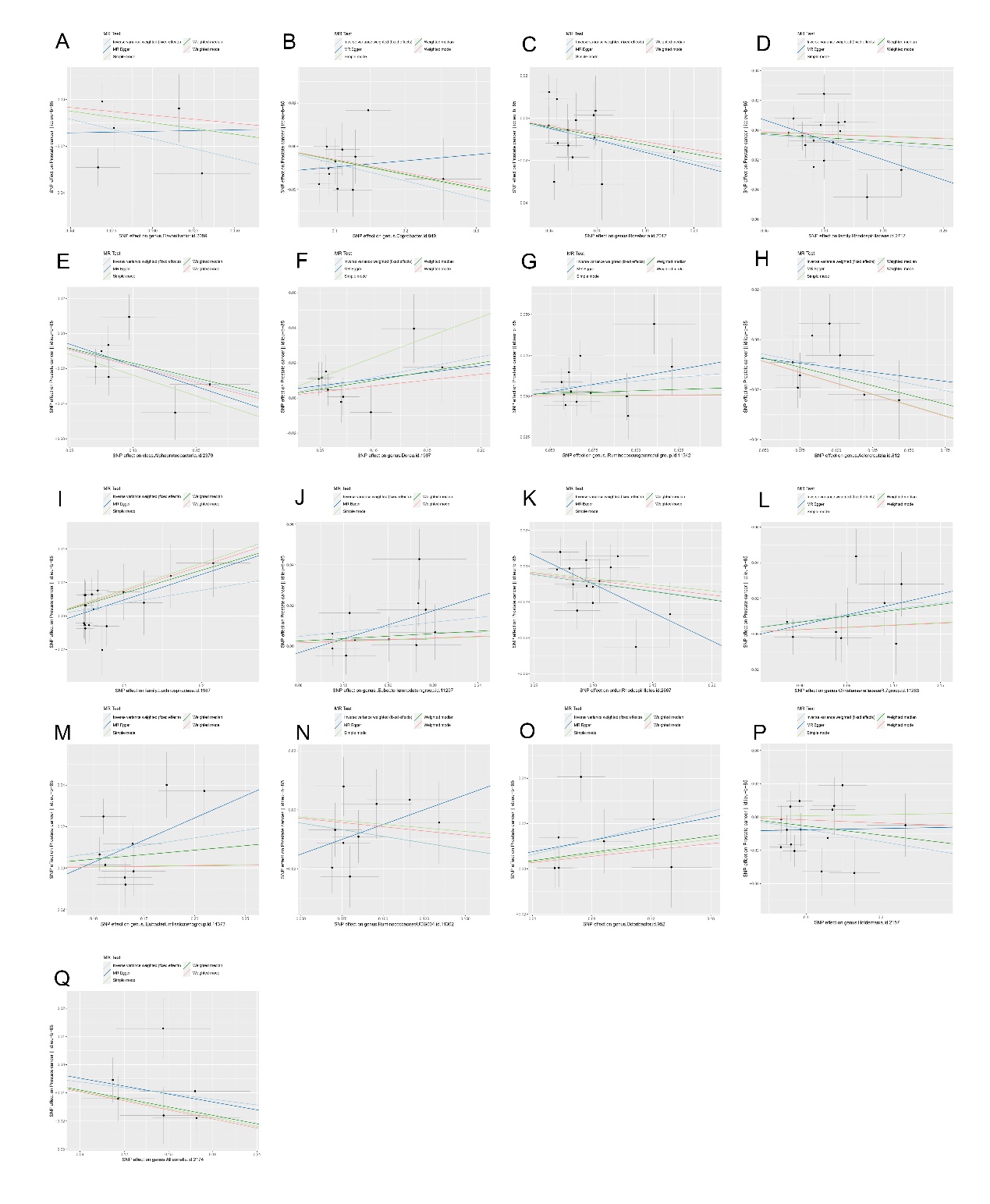


**Supplement Figure S2**

Scatter plots of significant and nominal significant estimates from genetically predicted

microbiotas(A) Genus.Flavonifractor; (B) Genus Coprobacter; (C) Genus Roseburia; (D) Family Rhodospirillaceae; (E) Class Alphaproteobacteria ; (F) Genus Dorea; (G) Genus Ruminococcus gauvreauii group; (H) Genus Adlercreutzia; (I) Family Lachnospiraceae;(J) Genus Eubacteriumnodatumgroup (K) Order Rhodospirillales;(L) Genus ChristensenellaceaeR 7group;(M) Genus Eubacteriumfissicatena group;(N) Genus RuminococcaceaeUCG004;(O) Genus Odoribacter;(P) Genus Holdemania;(Q) Genus Allisonella on prostate cancer.


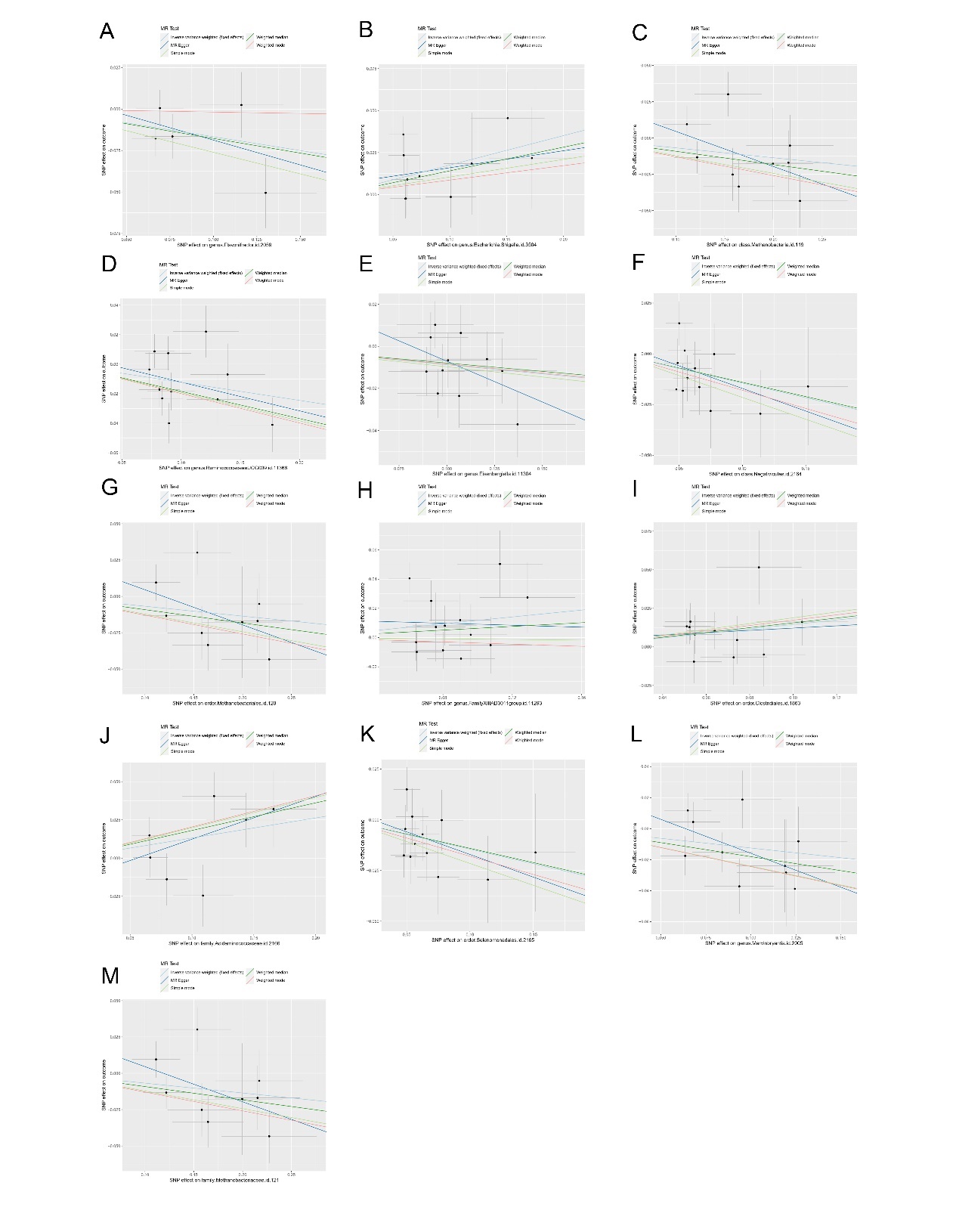


**Supplement Figure S3**

Scatter plots of significant and nominal significant estimates from genetically predicted

microbiotas(A) Genus Flavonifractor; (B) Genus Escherichia.Shigella (C) Class Methanobacteria (D) Genus Ruminococcaceae UCG009; (E) Genus Eisenbergiella ; (F) Class Negativicutes (G) Order Methanobacteriales; (H) Genus FamilyXIIIAD3011 group; (I) Order Clostridiales;(J) Family Acidaminococcaceae (K) Order Selenomonadales (L) Genus Marvinbryantia;(M) Family Methanobacteriaceae; on benign prostatic hyperplasia.

**
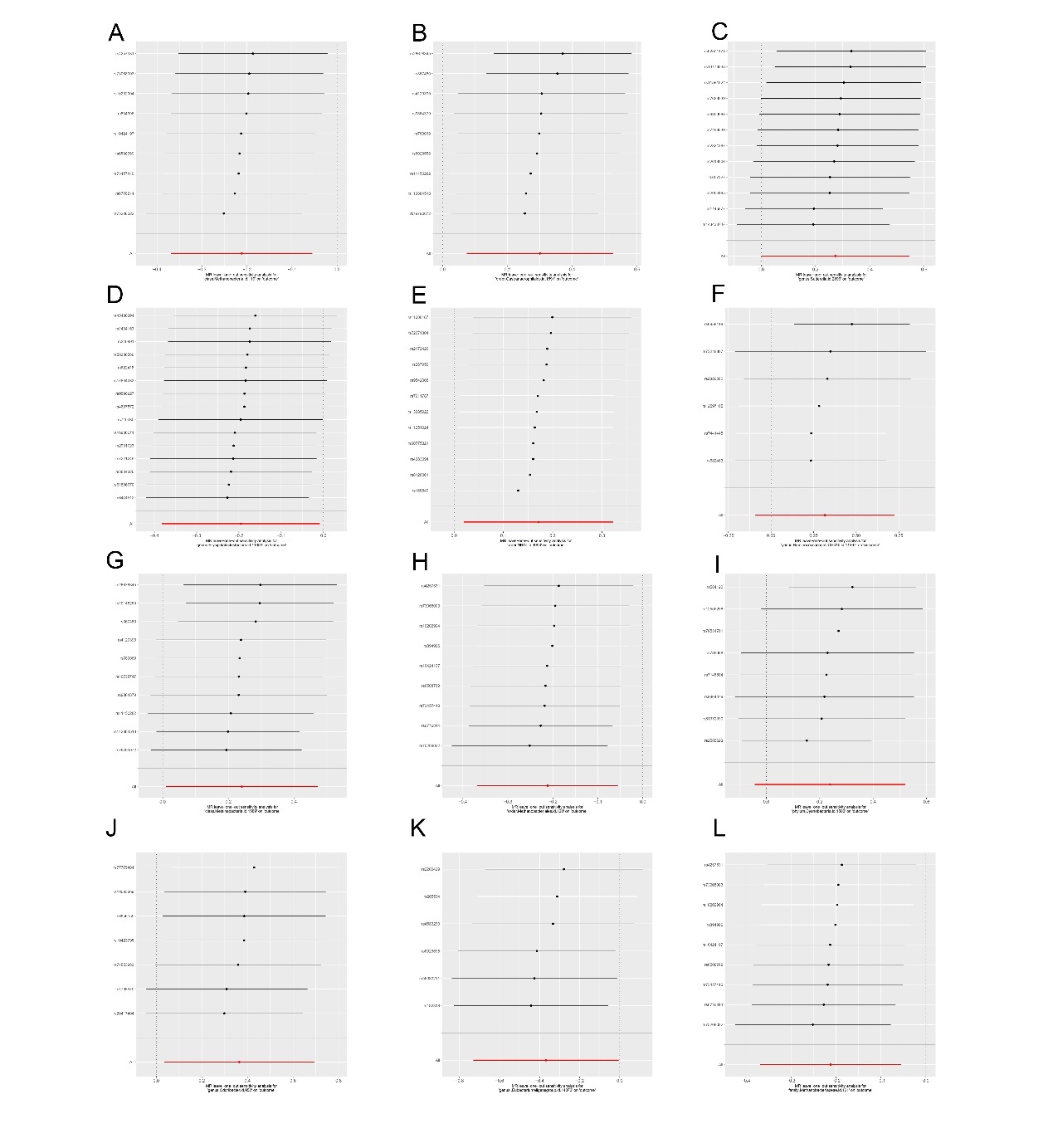
**

**Supplement Figure S4**

Leave-one-out plots of significant and nominal significant estimates from genetically predicted microbiotas (A) Family Methanobacteriaceae; (B) Order Gastranaerophilales; (C) Genus Sutterella; (D) Genus Erysipelatoclostridium; (E) Order NB1n ; (F) Genus RuminococcaceaeUCG010; (G) Class Melainabacteria; (H) Order Methanobacteriales; (I) Phylum Cyanobacteria;(J) Genus Odoribacter;(K) GenusEubacterium eligens group;(L) Family Methanobacteriaceae on prostatitis


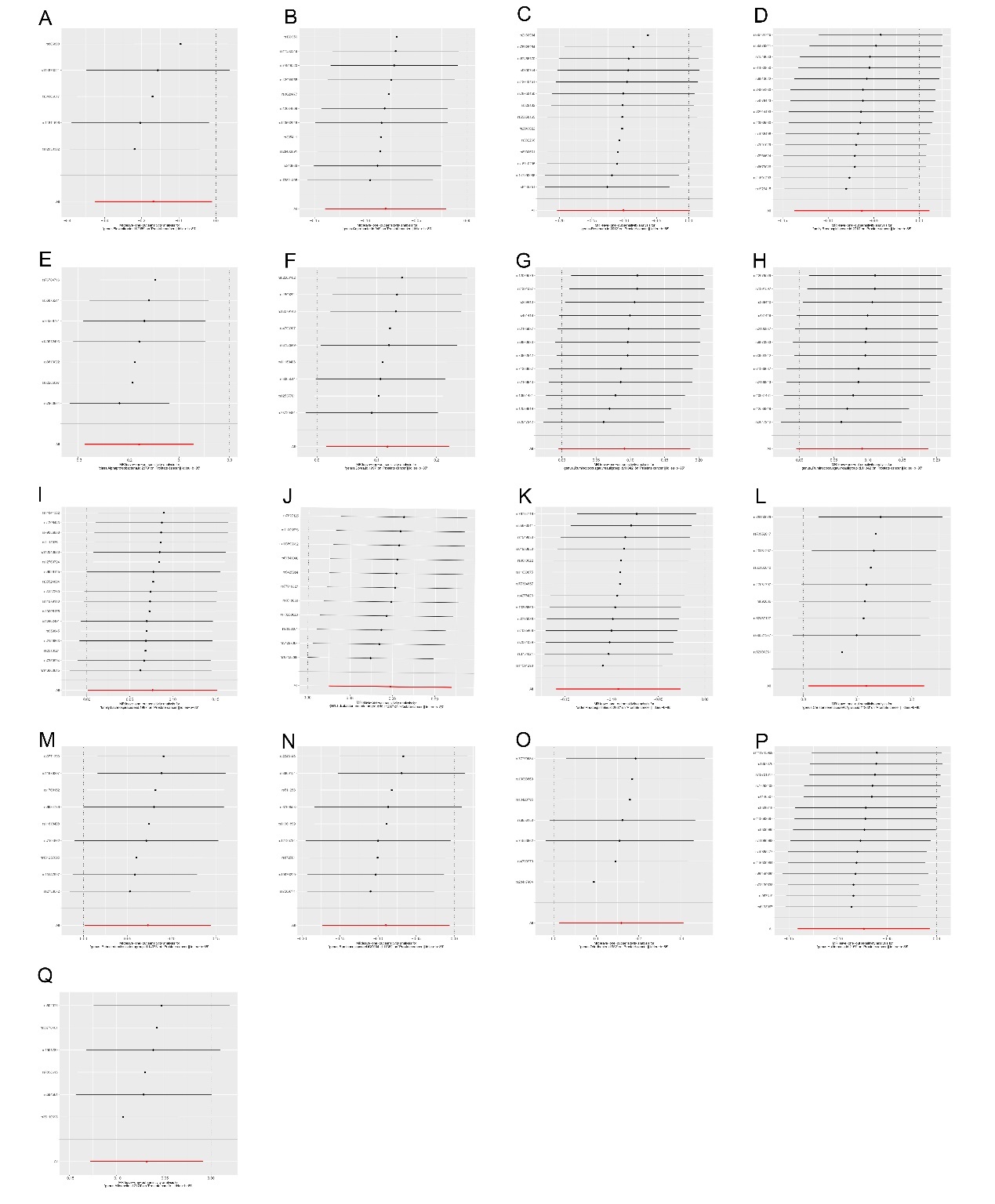


**Supplement Figure S5**

Leave-one-out plots of significant and nominal significant estimates from genetically predicted microbiotas (A) Genus.Flavonifractor; (B) Genus Coprobacter; (C) Genus Roseburia; (D) Family Rhodospirillaceae; (E) Class Alphaproteobacteria ; (F) Genus Dorea; (G) Genus Ruminococcus gauvreauii group; (H) Genus Adlercreutzia; (I) Family Lachnospiraceae;(J) Genus Eubacteriumnodatumgroup (K) Order Rhodospirillales;(L) Genus ChristensenellaceaeR 7group;(M) Genus Eubacteriumfissicatena group;(N) Genus RuminococcaceaeUCG004;(O) Genus Odoribacter;(P) Genus Holdemania;(Q) Genus Allisonella on prostate cancer.


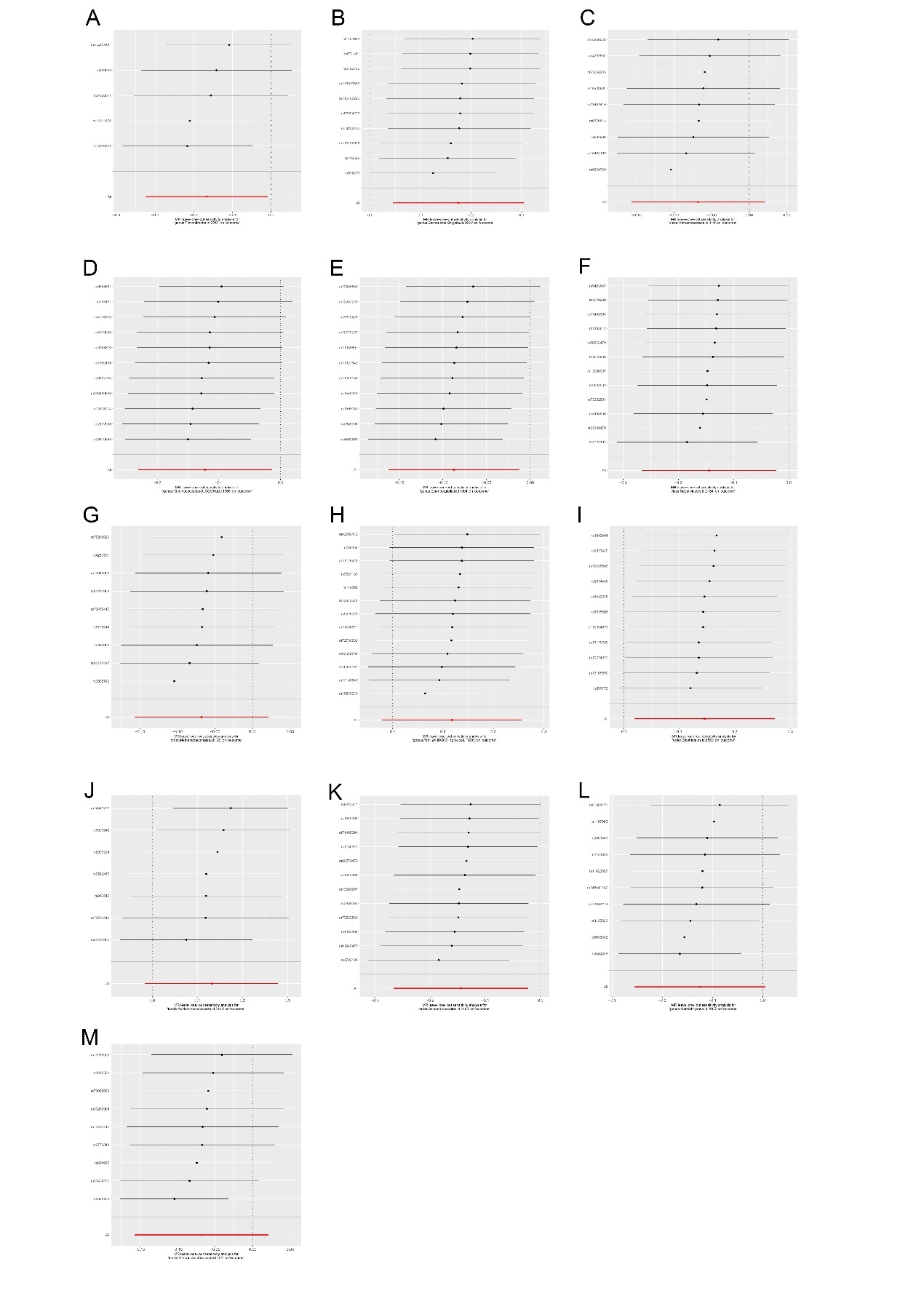


**Supplement Figure S6**

Leave-one-out plots of significant and nominal significant estimates from genetically predicted microbiotas (A) Genus Flavonifractor; (B) Genus Escherichia.Shigella (C) Class Methanobacteria (D) Genus Ruminococcaceae UCG009; (E) Genus Eisenbergiella ; (F) Class Negativicutes (G) Order Methanobacteriales; (H) Genus FamilyXIIIAD3011 group; (I) Order Clostridiales;(J) Family Acidaminococcaceae (K) Order Selenomonadales (L) Genus Marvinbryantia;(M) Family Methanobacteriaceae; on benign prostatic hyperplasia.
